# Increasing Use of Antenatal Magnesium Sulphate Prior to Preterm Birth for Preventing Cerebral Palsy in Australia and New Zealand, 2012–2020: A Binational Registry Study

**DOI:** 10.1111/ajo.13937

**Published:** 2025-01-16

**Authors:** Emily Shepherd, Sarah McIntyre, Alice Rumbold, Tasneem Karim, Shona Goldsmith, Amy Keir, Amanda Poprzeczny, Rod W. Hunt, Nadia Badawi, Christopher J. D. Mckinlay, Caroline A. Crowther, Lisa Yelland, the Australian and New Zealand Neonatal Network (ANZNN)

**Affiliations:** ^1^ Women and Kids Theme South Australian Health and Medical Research Institute (SAHMRI) Adelaide South Australia Australia; ^2^ Adelaide Medical School The University of Adelaide Adelaide South Australia Australia; ^3^ Cerebral Palsy Alliance Research Institute, Sydney Medical School The University of Sydney Sydney New South Wales Australia; ^4^ Women's and Children's Hospital Adelaide South Australia Australia; ^5^ Department of Paediatrics Monash University Melbourne Victoria Australia; ^6^ Monash Newborn Monash Children's Hospital Melbourne Victoria Australia; ^7^ Grace Centre for Newborn Intensive Care The Children's Hospital at Westmead Sydney New South Wales Australia; ^8^ Department of Paediatrics: Child and Youth Health The University of Auckland Auckland New Zealand; ^9^ Liggins Institute The University of Auckland Auckland New Zealand; ^10^ School of Public Health The University of Adelaide Adelaide South Australia Australia

**Keywords:** cerebral palsy, guideline adherence, infant, magnesium sulphate, premature, premature birth

## Abstract

We assessed the use of magnesium sulphate prior to preterm birth for preventing cerebral palsy in an Australian and New Zealand registry study. Use increased markedly from 32.3% (2012) to 78.8% (2020) (*p* < 0.001). Binational approaches to sustain and explore the feasibility of further increasing use, informed by evolving evidence and guidelines, are needed.

## 1 Introduction

Cerebral palsy remains the most common childhood physical disability. In Australia, one child in 700 is born with cerebral palsy [1]. Preterm birth, before 37 weeks' gestation, is a major risk factor; over 40% of children with cerebral palsy are born preterm versus approximately 8% of the general population [1]. Prevention is fundamental as there is no cure.

Antenatal magnesium sulphate was established as a preterm cerebral palsy prevention strategy in syntheses of randomised trials 15 years ago [2]. This year, a Cochrane review reaffirmed with high certainty that magnesium sulphate for preterm fetal neuroprotection reduces cerebral palsy (relative risk [RR] 0.71, 95% confidence interval [CI] 0.57–0.89), and death or cerebral palsy (RR 0.87, 95% CI 0.77–0.98) [3].

In 2010, National Health and Medical Research Council (NHMRC)‐endorsed Australian and New Zealand guidelines recommended magnesium sulphate administration prior to preterm birth before 30 weeks' gestation for fetal neuroprotection [4]; and in 2011, a three‐year implementation project ‘WISH’ (Working to Improve Survival and Health for babies born very preterm) commenced [5]. In 2012, the Australian and New Zealand Neonatal Network (ANZNN) commenced data collection on its use. The ANZNN is a network of 30 tertiary‐level Neonatal Intensive Care Units (NICUs) that monitors care of high‐risk infants [6].

Assessing magnesium sulphate use is important to evaluate the implementation of current recommendations. To date, use has been estimated by surveys or single‐centred cohorts, which have limitations [5, 7, 8]. Our primary aim was to assess Australian and New Zealand use of antenatal magnesium sulphate prior to preterm birth before 30 weeks' gestation over time. Our secondary aim was to explore whether use varied by maternal and birth characteristics.

## 2 Materials and Methods

We included live births before 30 weeks' gestation within the ANZNN registry from 1 January 2012–31 December 2020. Data on antenatal magnesium sulphate use over time were presented graphically. Univariable log‐binomial regression models explored associations between pre‐defined characteristics and magnesium sulphate use, with results presented as RR with 95% CI and 2‐sided *p* values. Data were analysed using Stata V.18.0. The study was approved by the Women's and Children's Health Network Human Research Ethics Committee (2022/HRE00259).

## 3 Results and Discussion

**FIGURE 1:**
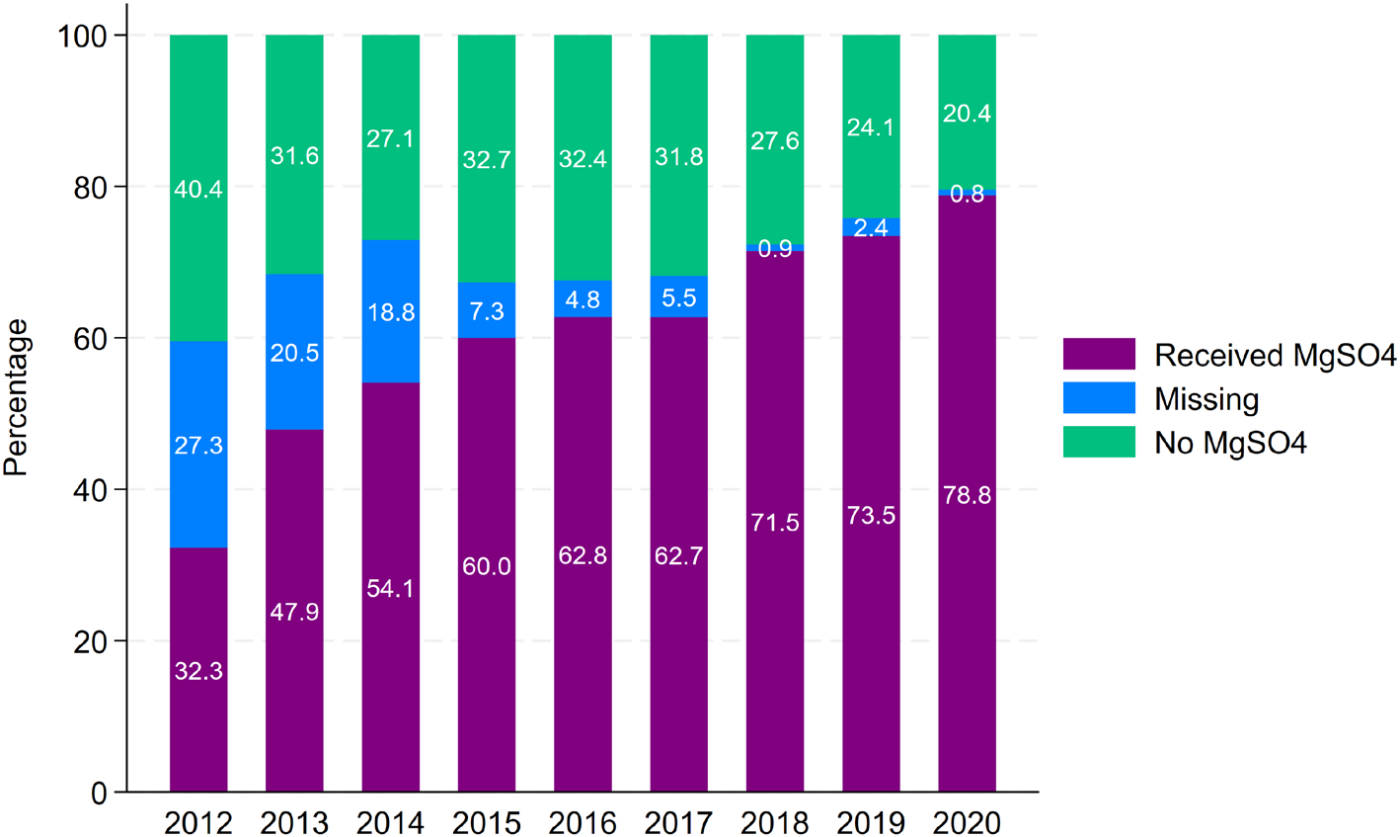
Use of magnesium sulphate among infants born before 30 weeks' gestation in Australia and New Zealand, 2012–2020 (*N* = 18394). MgSO_4_ = magnesium sulphate.

The cohort comprised 18394 infants (10114 [55%] males; 13677 [74.4%] singletons) born at 27 weeks median gestational age and 988 g median birthweight (Data S1, table). The percentage of infants exposed to antenatal magnesium sulphate increased from 32.3% (675/2092) in 2012 to 78.8% (1567/1988) in 2020 (*p* < 0.001)—an average annual increase of 5.8%. Consequently, the proportion of infants not exposed and with exposure ‘missing’ decreased (Figure 1; exposure under missing data assumptions in Data S1, figure).

Use of magnesium sulphate was most common among infants born to mothers who had assisted conception, presented with hypertension in pregnancy, or suspected growth restriction, received antibiotics and/or corticosteroids, were delivered at 24–27 weeks, and were in tertiary hospitals. Conversely, use was lowest among infants born to mothers who were younger, of Aboriginal, Māori, or Pacific Islander ethnicity, gave birth vaginally, or presented with antepartum haemorrhage or preterm labour. Usage was similar across single and multiple births (Table 1).

**TABLE 1:** Associations between maternal and birth characteristics and use of magnesium sulphate among infants born before 30 weeks' gestation in Australia and New Zealand, 2012–2020.

Characteristic	Magnesium sulphate given [*n*/*N* (%)]	Magnesium sulphate not given [*n*/*N* (%)]	Relative risk (95% CI)	*p*
Year of birth^a^				
1‐year increase	11087/16582 (66.9%)	5495/16582 (33.1%)	1.05 (1.04–1.05)	< 0.001
Maternal age				
< 20	412/718 (57.4%)	306/718 (42.6%)	0.84 (0.78–0.89)	< 0.001
20–24	1594/2470 (64.5%)	876/2470 (35.5%)	0.94 (0.91–0.98)	< 0.001
25–29	2803/4087 (68.6%)	1284/4087 (31.4%)	Reference	< 0.001
30–34	3371/5023 (67.1%)	1652/5023 (32.9%)	0.98 (0.95–1.01)	0.13
35–39	2135/3135 (68.1%)	1000/3135 (31.9%)	0.99 (0.96–1.02)	0.66
≥ 40	736/1064 (69.2%)	328/1064 (30.8%)	1.01 (0.96–1.06)	0.71
Assisted conception in this pregnancy				
No	9089/13847 (65.6%)	4758/13847 (34.4%)	Reference	
Yes	1410/1944 (72.5%)	534/1944 (27.5%)	1.11 (1.07–1.14)	< 0.001
Ethnicity of mother				
Caucasian	6969/10158 (68.6%)	3189/10158 (31.4%)	Reference	< 0.001
Aboriginal	717/1128 (63.6%)	411/1128 (36.4%)	0.93 (0.88–0.97)	0.001
Asian	1738/2478 (70.1%)	740/2478 (29.9%)	1.02 (0.99–1.05)	0.13
Māori	504/832 (60.6%)	328/832 (39.4%)	0.88 (0.83–0.93)	< 0.001
Pacific Islander	261/416 (62.7%)	155/416 (37.3%)	0.91 (0.85–0.99)	0.020
Other	431/626 (68.8%)	195/626 (31.2%)	1.00 (0.95–1.06)	0.90
Plurality				
Singleton	8260/12340 (66.9%)	4080/12340 (33.1%)	Reference	0.94
Twins	2605/3909 (66.6%)	1304/3909 (33.4%)	1.00 (0.97–1.02)	0.73
Higher order multiples	222/333 (66.7%)	111/333 (33.3%)	1.00 (0.92–1.08)	0.92
Presenting antenatal complication^b^				
Preterm labour	3958/6265 (63.2%)	2307/6265 (36.8%)	Reference	< 0.001
Preterm prelabour rupture of membranes	2494/3718 (67.1%)	1224/3718 (32.9%)	1.06 (1.03–1.09)	< 0.001
Antepartum haemorrhage	1016/1770 (57.4%)	754/1770 (42.6%)	0.91 (0.87–0.95)	< 0.001
Suspected intrauterine growth restriction	464/591 (78.5%)	127/591 (21.5%)	1.24 (1.19–1.30)	< 0.001
Fetal compromise	1057/1584 (66.7%)	527/1584 (33.3%)	1.06 (1.02–1.10)	0.007
Hypertension in pregnancy	1515/1741 (87%)	226/1741 (13%)	1.38 (1.34–1.41)	< 0.001
Other	577/900 (64.1%)	323/900 (35.9%)	1.01 (0.96–1.07)	0.58
Antibiotics within 48 h of birth				
No	4036/6867 (58.8%)	2831/6867 (41.2%)	Reference	
Yes	6626/8991 (73.7%)	2365/8991 (26.3%)	1.25 (1.22–1.28)	< 0.001
Antenatal corticosteroids for fetal lung enhancement				
No	198/1364 (14.5%)	1166/1364 (85.5%)	Reference	
Yes	10854/15140 (71.7%)	4286/15140 (28.3%)	4.94 (4.34–5.62)	< 0.001
Gestational age at birth (completed weeks)				
< 24	448/683 (65.6%)	235/683 (34.4%)	0.96 (0.91–1.02)	0.16
24–27	5831/8540 (68.3%)	2709/8540 (31.7%)	Reference	< 0.001
28–29	4808/7359 (65.3%)	2551/7359 (34.7%)	0.96 (0.94–0.98)	< 0.001
Place of birth				
Tertiary hospital	10484/14708 (71.28%)	4224/14708 (28.72%)	Reference	
Other	599/1859 (32.22%)	1260/1859 (67.78%)	0.45 (0.42–0.48)	< 0.001
Method of birth				
Vaginal birth	3699/5939 (62.28%)	2240/5939 (37.72%)	Reference	< 0.001
Vaginal instrumental birth	251/338 (74.26%)	87/338 (25.74%)	1.19 (1.12–1.27)	< 0.001
Caesarean section in labour	2601/3926 (66.25%)	1325/3926 (33.75%)	1.06 (1.03–1.10)	< 0.001
Caesarean section no labour	4519/6342 (71.26%)	1823/6342 (28.74%)	1.14 (1.12–1.17)	< 0.001

*Note:* For characteristics with > 2 categories, the *p*‐value for the reference category is from the global test of no association.

Abbreviation: CI = confidence interval.

^a^ Descriptives are *n*/*N* (%) for the full cohort.

^b^ Analysed using a log‐Poisson regression model with robust variance estimation due to non‐convergence of the log‐binomial regression model.

Our study is the first population‐based assessment of magnesium sulphate prior to preterm birth in Australia and New Zealand, and, to our knowledge, is the largest analysis to date. Exposure reached 78.8% in 2020. This is comparable to 83.1% reported in 2018–2020 in England during a national quality improvement intervention, where ‘imminent birth’ was the leading reason treatment was not given [9]. Our analyses also suggest unanticipated or precipitous preterm labour (e.g., where corticosteroids are also not given) may impede use compared with anticipated or planned birth (e.g., in the setting of growth restriction). We found use was low in births at non‐tertiary hospitals. While current guidance promotes use in these settings, it is contingent on ‘service capability and staffing,’ which may be less available to fulfil maternal and fetal monitoring requirements [4].

Given high‐certainty evidence of benefit [3], exploring feasibility to further increase binational use of magnesium sulphate is warranted. Due to the nature of preterm labour and birth, however, there is likely an ‘upper ceiling’ for attainable uptake. During a local quality improvement program, a South Australian tertiary hospital reached 86.0% uptake, though it noted, ‘without further ongoing implementation investment… rates are unlikely to reach and be maintained at 90%’ [8].

This study is based on robust, prospectively collected data from a large network of NICUs, but is limited to routinely collected information. Therefore, it is not possible to delineate use for pre‐eclampsia/eclampsia [10] nor reasons for ‘non‐use.’

The population‐level impacts of increased magnesium sulphate use—notably trends in cerebral palsy diagnoses—are the subject of our ongoing investigation. Our planned international individual participant data meta‐analysis [11] may also influence future guideline revision and implementation strategies.

In conclusion, Australian and New Zealand use of antenatal magnesium sulphate prior to birth before 30 weeks' gestation has increased markedly across 2012–2020, suggesting increasing uptake of recommendations. Binational approaches to sustain and explore the feasibility of further improving use, informed by evolving evidence and guidelines, are needed.

## Conflicts of Interest

The authors declare no conflicts of interest.

## Supporting information


Data S1.

## Data Availability

Data supporting this study cannot be made available due to ethical restrictions.
