# Supplementary material for: Increasing Use of Antenatal Magnesium Sulphate Prior to Preterm Birth for Preventing Cerebral Palsy in Australia and New Zealand, 2012–2020: A Binational Registry Study
Source: Aust N Z J Obstet Gynaecol. 2025 Jan 16;65(4):538–42. doi: 10.1111/ajo.13937 (PMC12668883; doi:10.1111/ajo.13937)
Supplement: Supplementary file 1 — Data S1. [file AJO-65-538-s001.docx]

**Table.** Characteristics of infants born before 30 weeks’ gestation in Australia and New Zealand included in analyses, 2012-2020

| **Total**  N=18394 | |
| --- | --- |
| Received magnesium sulphate | |
| Yes | 11087 (60.3%) |
| No | 5495 (29.9%) |
| Missing | 1812 (9.9%) |
| Year of birth | |
| 2012 | 2092 (11.4%) |
| 2013 | 1983 (10.8%) |
| 2014 | 2052 (11.2%) |
| 2015 | 2050 (11.1%) |
| 2016 | 2130 (11.6%) |
| 2017 | 2071 (11.3%) |
| 2018 | 1990 (10.8%) |
| 2019 | 2038 (11.1%) |
| 2020 | 1988 (10.8%) |
| Plurality | |
| Singleton | 13677 (74.4%) |
| Twins | 4353 (23.7%) |
| Triplets | 348 (1.9%) |
| Quads | 9 (0.0%) |
| Quintuplets | 5 (0.0%) |
| Missing | 2 (0.0%) |
| Sex | |
| Male | 10114 (55.0%) |
| Female | 8278 (45.0%) |
| Indeterminate | 2 (0.0%) |
| Gestational age (completed weeks) | 27 (26-28) |
| Gestational age (completed weeks) | |
| 22 | 23 (0.1%) |
| 23 | 723 (3.9%) |
| 24 | 1664 (9.0%) |
| 25 | 2084 (11.3%) |
| 26 | 2607 (14.2%) |
| 27 | 3075 (16.7%) |
| 28 | 3813 (20.7%) |
| 29 | 4405 (23.9%) |
| Birthweight (grams) | 988 (780-1215) |
| Place of birth† | |
| Tertiary hospital | 16180 (88.0%) |
| Non-tertiary hospital | 2030 (11.0%) |
| Home birth | 34 (0.2%) |
| Born before arrival | 133 (0.7%) |
| Missing | 17 (0.1%) |
| Method of birth | |
| Vaginal | 6629 (36.0%) |
| Instrument | 369 (2.0%) |
| Caesarean in labour | 4359 (23.7%) |
| Caesarean pre-labour | 6987 (38.0%) |
| Missing | 50 (0.3%) |

Note: Values in table are n (%) or median (interquartile range [IQR]). Denominator for all percentages is N=18394.

†Babies born in non-tertiary settings were admitted to tertiary-level Neonatal Intensive Care Units (NICUs) during their first 28 days of life and thus registered with the Australian and New Zealand Neonatal Network (ANZNN).

**Figure.** Use of magnesium sulphate among infants born before 30 weeks’ gestation in Australia and New Zealand under different assumptions about missing data (N=18,394)


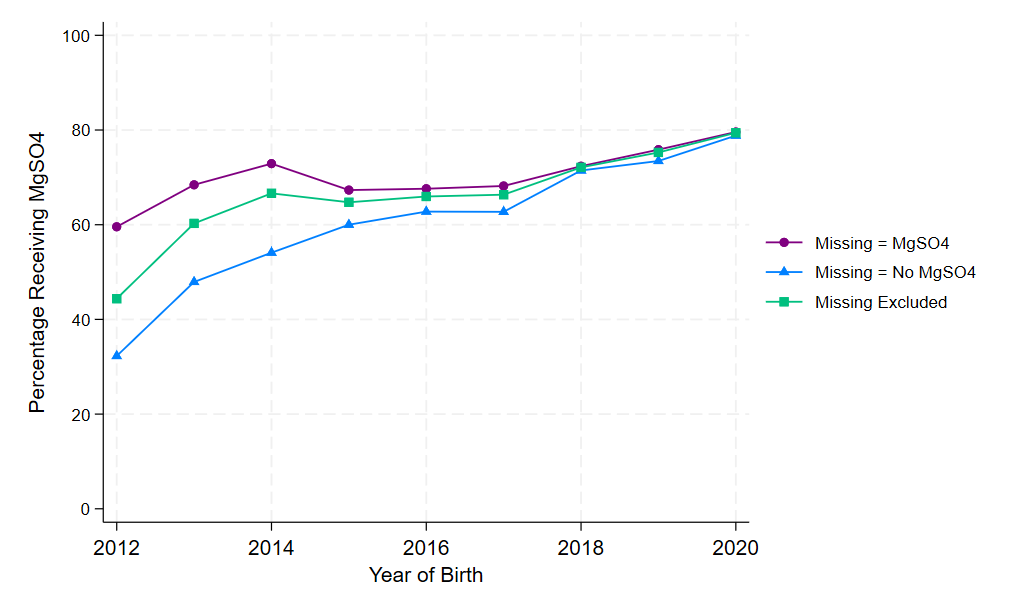


MgSO4 = magnesium sulphate.

Note: Plot shows the percentage of infants who received MgSO4 over time.

‘Missing = MgSO4’ assumes all infants with missing MgSO4 status received MgSO4 and represents the upper limit on the percentage.

‘Missing = No MgSO4’ assumes all infants with missing MgSO4 status did not receive MgSO4 and represents the lower limit on the percentage.

‘Missing Excluded’ is based on the subset of infants where MgSO4 status was available (N=16582) and represents the best estimate of the percentage under the assumption that the data are missing completely at random.
